# Diagnostic Value of Adenosine Deaminase and Its Isoforms in Type II Diabetes Mellitus

**DOI:** 10.1155/2016/9526593

**Published:** 2016-12-05

**Authors:** Bagher Larijani, Ramin Heshmat, Mina Ebrahimi-Rad, Shohreh Khatami, Shirin Valadbeigi, Reza Saghiri

**Affiliations:** ^1^Endocrinology and Metabolism Research Center, Endocrinology and Metabolism Clinical Sciences Institute, Tehran University of Medical Sciences, Tehran, Iran; ^2^Chronic Diseases Research Center, Endocrinology and Metabolism Population Sciences Institute, Tehran University of Medical Sciences, Tehran, Iran; ^3^Department of Biochemistry, Pasteur Institute of Iran, Tehran, Iran

## Abstract

*Background and Aims*. In the present study, we have investigated the activity of adenosine deaminase (ADA) as a diagnostic marker in type 2 (or II) diabetes mellitus (T2DM).* Design and Methods*. The deaminase activity of ADA1 and ADA2 was determined in serum from 33 patients with type 2 (or II) diabetes mellitus and 35 healthy controls. We also determined the proportion of glycated hemoglobin (HbA1c).* Results*. Our results showed significant differences between total serum ADA (tADA) and ADA2 activities in the diabetic groups with HbA1c < 8 (%) and HbA1c ≥ 8 (%) with respect to the values in healthy individuals (*p* < 0.001). ADA2 activity in patients with high HbA1c was found to be much higher than that in patients with low HbA1c (*p* = 0.0001). In addition, total ADA activity showed a significant correlation with HbA1c (*r* = 0.6, *p* < 0.0001).* Conclusions*. Total serum ADA activity, specially that due to ADA2, could be useful test for the diagnosis of type 2 (or II) diabetes mellitus.

## 1. Introduction

Diabetes mellitus is a common disorder of glucose homeostasis which grows epidemically. The number of people diagnosed with T2DM is estimated about 380 million by the World Health Organization. This number is expected to increase to 592 million people by the year 2035 [1]. Glycosylated hemoglobin (HbA1c) has been suggested by International Diabetes Federation (IDF) for diagnosis of diabetes. HbA1c is supposed to be the best test for long-term control of blood glucose [2]. Metabolic disturbance and immunological imbalance are two important key factors in type 2 diabetes mellitus. Insulin deficiency and insulin resistance are the most important metabolic factors in type 2 diabetes. Immunological disturbance in type 2 diabetic individuals has been associated with cell-mediated immunity and inappropriate T-lymphocyte function [3]. Adenosine deaminase (adenosine aminohydrolase, EC 3.5.4.4, ADA) is one of the key enzymes of purine nucleoside metabolism that catalyzes the deamination of adenosine and deoxyadenosine to inosine and deoxyinosine, respectively. ADA, an enzyme essential for the differentiation and proliferation of lymphocytes and monocyte, macrophage system, has been used for monitoring several immune system diseases. This enzyme was considered as a suitable marker of cell-mediate immunity. T cells are the recipient of adenosine signaling produced by B cells. Adenosine decreases the activation of T cells. ADA that exists on the surface of B lymphocytes could inactivate the adenosine signal. ADA is localized on the external surface of erythrocytes this might be in turn associated with inactivation of extracellular adenosine. Binding complexing protein on surface of fibroblasts has been considered to be a surface receptor for ADA [4, 5]. Adenosine deaminase includes two isoforms with unique biochemical properties. ADA1 can exist as a monomer with 30–40 KDa molecular weight or as a dimer with 280 KDa molecular weight while ADA2 is a 110 KDa protein [6, 7]. The ADA1 is found in all cells with the highest activity in lymphocytes and monocytes, whereas ADA2 is the predominant isoform in the serum of normal subjects. The major source of ADA2 is likely to be monocyte-macrophage system that produced it in response to pathogen factors and encoded by CECRI gene. ADA2 is a member of the new family of growth factors called ADCFs (ADA-related growth factors). ADA2 is strongly increased in inflammatory diseases such as rheumatoid arthritis and tuberculosis, AIDS, and diabetes [8–10]. Studies have shown that the protein attached to ADA1 molecule is identified to be CD26/DPPIV [11]. CD26 is a T cell activating antigen and transmembrane glycoprotein. On the surface of T-lymphocytes, ADA binds to CD26 via A2bR (adenosine A2B receptors) [12]. Moreover, CD26/DPPIV inactivates the incretin hormone glucagon-like peptide-1 (GLP-1) and glucose dependent insulin tropic polypeptide (GIP). DPPIV inhibitors stabilize endogenous GLP-1 at physiological concentration, induce insulin secretion in glucose-dependent manner and prevent the degradation of GLP-1 [13]. Recent studies show ADA increase in tissue of diabetic rats. ADA increased activity was reported in the lymph nodes and splenocyte of diabetes-prone BB rats [14]. Genetically, ADA locus in chromosome 20 is associated with locus type 2 diabetes [15]. Adenosine deaminase is an important enzyme for modulating adenosine concentration. Adenosine stimulates insulin activity via several processes such as glucose transport, lipid synthesis, pyruvate dehydrogenase activity, and Leucine oxidation. Adenosine plays an important role in bioactivity of insulin and regulates insulin activity in various tissues such as liver, myocardium white adipose, and skeletal muscles [16, 17]. Adenosine potentiates the action of insulin in myocardium and adipose tissue and inhibits liver and skeletal muscle. Adenosine mimics the action of insulin on glucose and lipid metabolism in adipose tissue [18]. Adenosine has antilipolysis property in adipose tissue and increases insulin sensitivity for glucose transport [19]. Adenosine increases accessibility about 25% of GLUT4 to cell surface for glucose transportation [20]. Adenosine with binding to A1 receptor increases the accumulation of the insulin-induced PIP3 and PKB in postreceptor phase [21]. Adenosine has also been shown to increase gluconeogenesis and glycogenolysis via increasing cyclic AMP (CAMP) by stimulation of hepatic adenylate cyclase through adenosine A2a receptor binding in liver. Both or either of these actions causes the increase of local insulin resistance and glucose output from the liver [22]. In this study we have investigated the alternation of serum ADA activities and its mechanism in type 2 diabetic patients.

## 2. Material and Method

### 2.1. Patients

33 patients with type 2 diabetes who referred to Diabetes Center of Shariati Hospital were diagnosed by the endocrinologist and their blood was drawn during 6 months. No patients displayed any kind of infection or inflammatory diseases which lead to increase in ADA. This group was divided into two groups according to hemoglobin A1c (HbA1c) level, namely, patients whose HbA1c levels were less than 8 and those with HbA1c level more than 8. Blood was also drawn from 35 healthy individuals as the control subjects.

### 2.2. Blood Sampling Protocol

#### 2.2.1. Serum Samples

Blood samples were obtained from patients and healthy subjects after 12 h fasting. Fasting blood samples were collected to centrifugation at 3,000 rpm for 10 min at 4°C to obtain serum. All samples were stored at −80°C after separation.

#### 2.2.2. Measurement of Adenosine Deaminase

The ADA activity was assayed by the ADA kit of Diazyme Laboratories and with model 912 type Autoanalyzer (Hitachi Co. Ltd., Tokyo, Japan). The ADA assay is based on enzymatic deamination of adenosine to inosine in this kit. Inosine is converted to hypoxanthine by purine nucleoside phosphorylase. Hypoxanthine is then converted to uric acid and hydrogen peroxide (H_2_O_2_) by xanthine oxidase. H_2_O_2_ is further reacted with N-ethyl-N-(2-hydroxy-3-sulfopropyl)-3-methylaniline and 4-aminoantipyrine in the presence of peroxidase to generate quinone dye which is monitored in a kinetic manner.

To distinguish between the ADA1 and ADA2 forms, the ADA activity was measured using the same technique with and without EHNA (erythro-9-(2-hydroxy-3-nonyl) adenine), obtained from Sigma-Aldrich (St. Louis, MO, USA). EHNA, a potent selective inhibitor of ADA1, was used at final concentration of 0.1 mmol/L. In its presence, only the ADA2 isoform is active. The ADA1 activity is then calculated by subtracting the ADA2 activity from the total ADA activity.

### 2.3. Assessment of Glycated Hemoglobin (HbA1c)

Diazyme direct enzymatic HbA1c analysis was evaluated on the Hitachi 912 Autoanalyzer (Hitachi Co. Ltd., Tokyo, Japan) using blood samples. We have applied simple enzymatic assay for HbA1C using neutral protease and FPOX. Preparation of samples was carried out by mixing 500 *μ*L of lysis buffer with 25 *μ*L of whole blood and incubating for 10 min at room temperature.

### 2.4. Statistical Analysis

All results were expressed in terms of mean ± standards deviation (SD). Data were analyzed by Package for Social Sciences (SPSS) version 16. Statistical differences between patient groups and controls were performed by one-way ANOVA test. Correlation between the ADA and HbA1c was measured by means of Pearson's Correlation Coefficient (*r*). HbA1c was expressed as a percentage. BMI was calculated as weight (kg)/height^2^ (m^2^). *p* value < 0.01 was considered statically significant.

## 3. Results

This study was carried out on 16 patients with HbA1c ≥ 8 who were designated poorly controlled DM and 17 patients with HbA1c < 8 who were well controlled and 35 healthy individuals as control subjects. Mean ± SD values of tADA, ADA1, and ADA2 were found in healthy subjects. Results are shown in Table 1.

The increasing of tADA, ADA1, and ADA2 activity in subjects with HbA1c < 8 and those with HbA1c ≥ 8 is significant in comparison with healthy ones (*p* < 0.001).

Anthropometric measurements and clinical parameters of the study subjects are shown in Table 2. As illustrated in the table, serum ADA activity in T2DM patients with HbA1c high and low was significantly higher than healthy group. These variations specially increased ADA2 in HbA1c ≥ 8 in comparison with HbA1c < 8 is investigated to be more.

**Figure 1:**
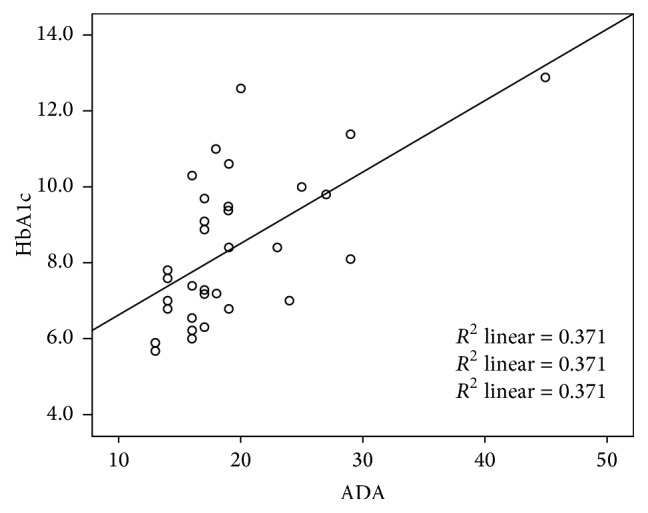
The correlation between HbA1c and tADA in diabetic patients.

As shown in Figure 1, we also observed significant positive correlation between serum ADA activities and HbA1c (*r* = 0.6, *p* < 0.0001).

## 4. Discussion

Type 2 diabetes is accompanied with collection of clinical and biochemical disorders which have been called metabolic syndrome X. These disorders include center obesity, hypertension, atherosclerosis, hypertriglyceridemia, increased cholesterol and LDL, and decreased HDL. The action of cytokines on the brain, liver, endothelium, and adipose tissue is a major factor of metabolic syndrome X. Cytokines stimulate the acute-phase proteins. In the short term, the acute-phase protein has survival values and regulates homeostasis and in long-term produces diseases [23]. These cytokines such as IL-1, IL-6, and TNF-*α* are produced from monocytes, macrophage, and adipose tissue. Insulin resistance and hyperglycemia increase the effect of cytokines on the liver and cause the secretion of IL-6 and TNF-*α* from monocytes and macrophage [24]. On the other hand, production of cytokines from monocytes and macrophage and the increase in acute-phase proteins elevate insulin resistance [25]. Aging, certain dietary components, smoking, and obesity are important factors in cytokine increase and immunity disturbance in type 2 diabetes. Immunity disturbance does not happen in all T2DM individuals and other major factors such as genetics, cytokine sensitivity, and acute-phase response contribute to its existence. Cytokine imbalance effects ADA activity [26]. Variation in cytokines especially cytokines which were produced by Th1 cells is associated with the increase in ADA serum activity. It also activates monocyte-macrophage cell system [27]. Defect in insulin activity required for T-lymphocytes in diabetes leads to abnormal T-lymphocyte proliferation and enhanced ADA activity [28]. The effects of ADA on T-lymphocytes and cytokines represent it as a suitable marker of cell-mediated immunity. The increased ADA levels in inflammatory and autoimmune diseases such as rheumatoid arthritis, tuberculosis, and systemic lupus erythematosus (SLE) make its role more significant. Likewise, immune system and increase and concentration of extracellular adenosine are considered as other major factors in ADA increase. Adenosine is a local hormone which regulates many biological activities. Adenosine causes coronary vasodilatation, bradycardia, inhibition of platelet aggregation, renal vasoconstriction, and regulation of channel ion activity. These processes are carried out via adenosine receptors (A1, A2a, A3, and A2b). In normal conditions, adenosine contains low concentration lesser than 1 *μ*m and is risen under metabolically conditions such as stress and tissue injuries to 4–10 *μ*m [29]. Extracellular adenosine concentration is regulated by two mechanisms of transportation of adenosine across the cell membrane and enzymatic regulation of adenosine concentration and can be activate adenosine receptors [9]. There exist two types of nucleoside transporters across the plasma membrane, the equilibrative facilitated-diffusion transport (ENT) and concentrative Na^+^ dependent transporters (CNT). Inhibition of these transporters potentiates the action of adenosine. Enzymatic regulation of adenosine concentration in mammals is dependent upon activity of 5-nucleotidase and two utilizing enzymes: adenosine deaminase and adenosine kinase [30]. Investigations on diabetic individuals show the increase in the value of adenosine. Reduction in the activity of adenosine kinase is a major factor in the increase of adenosine in these patients [31]. In addition, variation in adenosine receptors and transporters can change tissue sensitivity to adenosine [32–34]. Studies on adenosine concentration and its effect on cell functions show the important role of adenosine in glucose metabolism. In the range of 0.003–0.5 *μ*mol/kg, adenosine lowers the serum fatty acids and serum insulin. On the other hand, in the range of 0.5–50 *μ*mol/kg, its effect on hepatic A2a receptors stimulates gluconeogenesis and the increase of serum glucose [35]. The increase of adenosine level with decrease in adenosine kinase in diabetes results in deamination of adenosine and the increase in adenosine deaminase. In fact, the increase in ADA activity is a protective mechanism against adenosine elevation [26]. When the adenosine concentration is strongly elevated, ADA enzyme catalyzes deamination of adenosine via ADA1 and ADA2 (ADA-related growth factors) isoforms and decreases its concentration. Since the ADA2 increases CD4-T cells, it can be useful in stimulating immune system [10]. T-lymphocytes abnormal proliferation, increased secretion cytokines, and increased extracellular adenosine are major factors in the elevation of ADA. The increase of ADA in diabetic patients leads to metabolic changes of insulin especially in adipose tissues. ADA in adipose tissue causes the increase in lipolysis, the augmentation of hyperlipidemia, and the disturbance in antilipolysis activity. High concentration of fatty free acids (FFA) derived from lipolysis elevation causes oxidative phosphorylation and ATP retention in adipocytes [36]. ADA impairs PKB and PI3P production in insulin postreceptor phase and reduces insulin sensitivity in adipocytes [20]. Moreover, endogenic reduction of adenosine by ADA decreases insulin ability for the activation of tyrosine kinase receptor in submaximal concentration of insulin. Therefore, diabetic adipocyte cells require more insulin concentration [21]. ADA reduces GLUT4 accessibility to cell surface for glucose transporters [19]. Adenosine deamination is guided toward hypoxanthine uric acid production and xanthine oxidase enzyme in this process causes superoxides production. The increase membrane peroxidation alters Na^+^, K^+^, ATPase activity, and transport of metabolites across the membrane. The increase and production of superoxides enhance the risk of cardiovascular diseases in diabetic patients. ADA causes the high rate of uric acid which shows significant correlation between the ADA levels and uric acids in diabetics [21, 37]. On the other hand, high glucose level causes the increase in ADA attached DPPIV protein. Accordingly, the increase in glucose of diabetics leads to high DPPIV-ADA and increase in DPPIV-ADA results in incretin reduction and insulin secretion. Insulin deficiency and irregulation of glucose are major factors in the elevation of ADA in diabetes. Therefore, insulin can be a good quality way of ADA reduction. Insulin reduces ADA activity in diabetic tissues and regulates local concentration of adenosine. Some herbal drugs can reduce ADA and control blood glucose [38]. The ADA-CD26/DPPIV interaction is inhibited by the cell surface glycoprptein (gp120) in HIV1-infected individuals and it might result in increasing levels of serum ADA. ADA-CD26/DPPIV complex plays a main role in the regulation of immune activity and cell adhesion [39, 40]. ADA alongside other immunomodulatory enzymes acts as a deconstructive oxidative marker in diabetes and plays an important role in progression of its complications. ADA increase, especially ADA2, may serve as an immunoenzyme marker in the pathology of type 2 diabetes mellitus.

## Figures and Tables

**Table 1:** ADA and its isoforms activities in T2DM patients based on low and high HbA1c comparison with healthy controls.

Groups		tADA	ADA2	ADA1
		(IU/L)	(IU/L)	(IU/L)
Low HbA1c	Mean	16.18	9.41	6.76
	*N*	17	17	17
	Std. deviation	2.67	3.74	1.67
	Minimum	13	6	2.00
	Maximum	24	22	8.00
	Variance	7.15	14.00	2.81

High HbA1c	Mean	22.44	13.94	8.50
	*N*	16	16	16
	Std. deviation	7.420	5.19	3.96
	Minimum	16	8	4.00
	Maximum	45	24	22.00
	Variance	55.06	26.99	15.73

Control	Mean	14.00	7.66	6.34
	*N*	35	35	35
	Std. deviation	1.680	1.73	.48
	Minimum	11	5	6.00
	Maximum	17	11	7.00
	Variance	2.82	2.99	.23

**Table 2:** Anthropometric measurements and clinical parameters of T2DM patients and healthy control.

Characteristics	T2DM (*n* = 33)	Controls (*n* = 35)	*p* value
Age (years)	60.2 ± 8.7	65.8 ± 9.2	0.0008
BMI (kg/m^2^)	29.5 ± 7.00	21.3 ± 6.78	0.005
FPG (mg/dL)	156.7 ± 10.3	92.6 ± 7.58	<0.0001

Data are shown as the means ± SD. BMI, body mass index; T2D, type 2 diabetes mellitus; FPG, fasting plasma glucose; *p* < 0.001, ANOVA test analysis.

## Abbreviations

HbA1c: Glycated hemoglobin
T2DM: Type II diabetes mellitus
ADA: Adenosine deaminase
DPPIV: Dipeptidyl peptidase 4
PIP3: Phosphatidylinositol-trisphosphate
PKB: Protein kinase B
AMP: Adenosine monophosphate
HDL: High-density lipoprotein
LDL: Low-density lipoprotein
IL: Interleukin
TNF-*α*: Tumor necrosis factor alpha
FPOX: Fructosyl peptide oxidase
ADGFs: Adenosine-related growth factors.
